# Semi-automatic conversion of BioProp semantic annotation to PASBio annotation

**DOI:** 10.1186/1471-2105-9-S12-S18

**Published:** 2008-12-12

**Authors:** Richard Tzong-Han Tsai, Hong-Jie Dai, Chi-Hsin Huang, Wen-Lian Hsu

**Affiliations:** 1 Department of Computer Science & Engineering, Yuan Ze University, Chung-Li, Taiwan, R.O.C; 2 Institute of Information Science, Academia Sinica, Nankang, Taipei, Taiwan, R.O.C; 3 Department of Computer Science, National Tsing-Hua University, Hsinchu, Taiwan, R.O.C

## Abstract

**Background:**

Semantic role labeling (SRL) is an important text analysis technique. In SRL, sentences are represented by one or more predicate-argument structures (PAS). Each PAS is composed of a predicate (verb) and several arguments (noun phrases, adverbial phrases, etc.) with different semantic roles, including main arguments (agent or patient) as well as adjunct arguments (time, manner, or location). PropBank is the most widely used PAS corpus and annotation format in the newswire domain. In the biomedical field, however, more detailed and restrictive PAS annotation formats such as PASBio are popular. Unfortunately, due to the lack of an annotated PASBio corpus, no publicly available machine-learning (ML) based SRL systems based on PASBio have been developed. In previous work, we constructed a biomedical corpus based on the PropBank standard called BioProp, on which we developed an ML-based SRL system, BIOSMILE. In this paper, we aim to build a system to convert BIOSMILE's BioProp annotation output to PASBio annotation. Our system consists of BIOSMILE in combination with a BioProp-PASBio rule-based converter, and an additional semi-automatic rule generator.

**Results:**

Our first experiment evaluated our rule-based converter's performance independently from BIOSMILE performance. The converter achieved an F-score of 85.29%. The second experiment evaluated combined system (BIOSMILE + rule-based converter). The system achieved an F-score of 69.08% for PASBio's 29 verbs.

**Conclusion:**

Our approach allows PAS conversion between BioProp and PASBio annotation using BIOSMILE alongside our newly developed semi-automatic rule generator and rule-based converter. Our system can match the performance of other state-of-the-art domain-specific ML-based SRL systems and can be easily customized for PASBio application development.

## Background

The amount of biomedical literature available online continues to grow rapidly today, creating a need for automatic processing using bioinformatics tools. Many information extraction (IE) systems incorporating natural language processing (NLP) techniques have been developed for use in the biomedical field. A key IE task in this field is the extraction of relations between named entities (NEs), such as protein-protein and gene-disease interactions.

**Figure 1:**
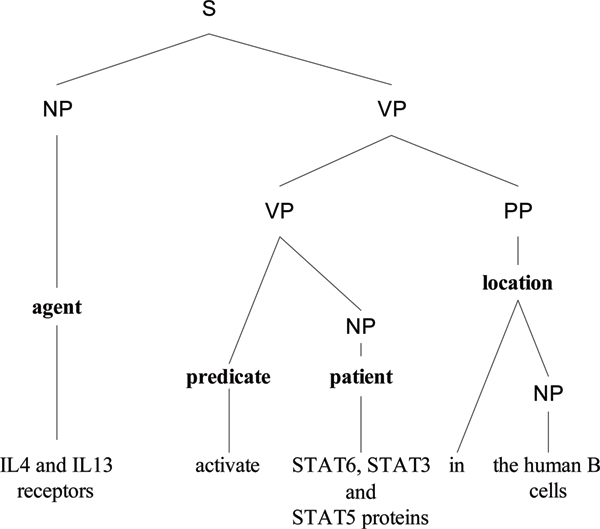
A parse tree annotated with semantic roles.

*Semantic role labeling *(*SRL*), also called shallow semantic parsing [1], is a popular semantic analysis technique for extracting relations. In SRL, sentences are represented by one or more *predicate-argument structures *(*PAS*), also known as propositions [2]. Each PAS is composed of a predicate (e.g., a verb) and several arguments (e.g., noun phrases) that have different semantic roles, including main arguments such as an agent that deliberately performs an action (e.g., **Bill **drank his soup quietly) and a patient that experiences an action (e.g., the falling rocks crushed **the car**), as well as adjunct arguments, such as time, manner, and location. Here, the term *argument *refers to a syntactic constituent of the sentence related to the predicate; and the term *semantic role *refers to the semantic relationship between a predicate (e.g., a verb) and an argument (e.g., a noun phrase) of a sentence. For example, in Figure 1, the sentence "IL4 and IL13 receptors activate STAT6, STAT3, and STAT5 proteins in the human B cells" describes a molecular activation process. It can be represented by a PAS in which "activate" is the predicate, "IL4 and IL13 receptors" comprises the agent, "STAT6, STAT3, and STAT5 proteins" comprises the patient, and "in the human B cells" is the location. Thus, the agent, patient, and location are the arguments of the predicate.

An important preliminary task in SRL is to define the set of possible semantic roles for each verb sense, referred to as a *roleset*. A roleset can be paired with a set of syntactic frames that shows all the acceptable syntactic expressions of those roles. This is called a *frameset *[3]. In 2000, the Proposition Bank project (PropBank) [3] published a guide, PropBank I [4,5], which defined a format for PAS annotation. Alongside PropBank I, the project also released a corpus of PAS's for 3,325 verbs in the newswire domain to facilitate ML-based SRL system development [6]. The semantic arguments of individual verbs in the PropBank I annotation are numbered from 0. For a specific verb, Arg0 is usually the argument corresponding to the agent [7], while Arg1 usually corresponds to the patient. However, higher-numbered arguments, which occupy about 10% of the total arguments, have no consistent role definitions. In addition to numbered arguments, there are also ArgMs, which refer to annotation of modifiers. (Detailed descriptions of all semantic role argument categories can be found in Additional file 1.) The semi-regular and flexible assignment of numbered arguments to semantic roles found in PropBank I facilitates formulation of the SRL task as a classification problem with machine-learning (ML) based systems. That is, given a phrase, the sentence containing it, and the predicate, a system must classify the phrase's semantic role corresponding to the predicate.

For specific applications, however, the flexible argument assignment of PropBank I annotation may be a disadvantage. In some cases, developers may wish to limit the semantic roles of each argument. Take the frameset of "delete" for example. Table 1 shows the frameset definition.

**Table 1:** Frameset of verb "delete" in PropBank I and PASBio

Predicate: delete		
Argument	PropBank I	PASBio

Arg0	entity removing	causer mechanism
		//mutation, alternative splicing//
Arg1	thing being removed	entity being removed
		//exon, gene, chromosomal region, cell//
Arg2	removed from	resultant product
		//transcripts//

As you can see in Table 1, the agent is defined as "entity removing", and the patient is defined as "thing being removed" in PropBank I. However, in certain biomedical events, a developer might want to limit the agent to being a certain causal mechanism such as a mutation or alternative splicing and the patient to being an "exon, gene, chromosomal region, [or] cell".

An alternative to PropBank, the PASBio [8] project provides more detailed and restrictive framesets for 29 biomedical verbs. The well-known biomedical text mining researchers Cohen and Hunter [9] have found the PASBio annotation viable for representing the PAS's of biomedical verbs. Several applications have been developed based on PASBio or following its spirit. For example, Shah et al. [10] used the frameset definitions of PASBio to construct semantic patterns which can extract information about tissue-specific gene expression from biomedical literature. Later, Shah and Bork applied this approach to construct the LSAT (Literature Support for Alternative Transcripts) database system [11]. Kogan et al. [12] followed the PASBio annotation to built a domain-specific set of PASs for the medical domain, which successfully extended PASBio to clinical texts. All these systems mainly use handcrafted rules to identify and classify arguments into semantic roles.

Unfortunately, due to the lack of an annotated corpus and inconsistent definitions between specific numbered arguments, no publicly available ML-based SRL systems based on the PASBio standard have been developed.

To be able to apply ML to the biomedical SRL problem, we constructed a biomedical domain specific proposition bank based on the more consistent PropBank I annotation format. The project, BioProp [13], defined roles for 30 common biomedical verbs and provided an annotated corpus on which we developed an ML-based SRL system, BIOSMILE [14]. This work was expanded upon with the release of our web-based search application, BIOSMILE web search [15], in February 2008.

In this paper, we aim to build a bridge between BioProp and PASBio to facilitate PASBio-based SRL system development. Using our system, one will first be able to roughly classify arguments' semantic roles according to BioProp, and then translate the PAS's into PASBio annotation using a rule-based converter.

## Methods

The approaches applied in this work include: (1) named entity tagging, (2) semantic role labeling following BioProp's annotation format, and (3) rule-based conversion from BioProp to PASBio annotation.

### Named entity tagging

According to our observations, some BioProp arguments are equivalent to other PASBio arguments only under certain conditions, usually defined as the presence of a certain named entity (NE) in a certain argument. For example, Arg1 of the verb "express" must be a gene or gene product in PASBio. Therefore, it is necessary to first tag all NEs in the sentences. To do this, we employ our previously developed NE recognition software, NERBio [16,17], to tag five NE types: protein, DNA, RNA, cell line, and cell type. We use a dictionary to find other NE types, such as extron and intron.

### Semantic role labeling

Before conversion to the PASBio annotation format, a fundamental step is to identify the PAS's of each sentence and annotate them using the BioProp format. Here, we briefly introduce how we constructed the BioProp-based SRL system, BIOSMILE, used for this task.

The first step was to construct a training corpus. In our previous work, Chou et al. [13], we annotated PAS's in GENIA's corpus of full parse trees, the GENIA Treebank (GTB) [18], using PropBank I framesets. We then defined and added framesets for biomedical verbs to fit specific usages in biomedical literature. However, all the new and modified framesets still conform strictly to the PropBank annotation format. A total of 2,304 PAS's were annotated for 49 biomedical verbs.

The second step we took was to formulate the SRL problem as an ML-based sentence tagging problem. The basic units of a sentence can be words, phrases, and constituents (nodes on a full parse tree). Punyakanok et al. [19] has shown that constituent-by-constituent (C-by-C, or node-by-node) tagging is the best formulation for the SRL problem; therefore, we adopted this formulation.

Finally, we constructed a biomedical full parser based on the Charniak parser [20] with GTB as its training data which could automatically generate parse trees for sentences. Its performance is reported in Additional file 1.

Using BioProp as the training corpus, C-by-C formulation, and the parse trees generated by our biomedical full parser, we then constructed our SRL system, BIOSMILE, following the maximum entropy ML model [21]. Details of the features used in our SRL system can be found in [14].

### Development of conversion rules

There are two main differences between BioProp and PASBio PAS framesets annotations: (1) PASBio developers usually define framesets to represent specific biological events. Therefore, for each argument, it is necessary to include information in addition to its semantic role, such as whether the argument should be a specific NE or contain specific keywords. (2) The order of arguments for a given verb sense in a BioProp frameset may not match that in a corresponding PASBio frameset. To deal with these two differences, we build conversion rules verb by verb using our semi-automatic rule-generation tool which describe under which conditions each mapping is valid. The algorithm used by the rule-generator compares corresponding framesets for a given verb sense, checks each argument in its PASBio frameset, and tries to find an argument in its BioProp frameset that has the same semantic role under a set of conditions. When a match is found, the algorithm maps a link between the two frameset arguments, which includes a description of required conditions (NEs and keywords).

**Figure 2:**
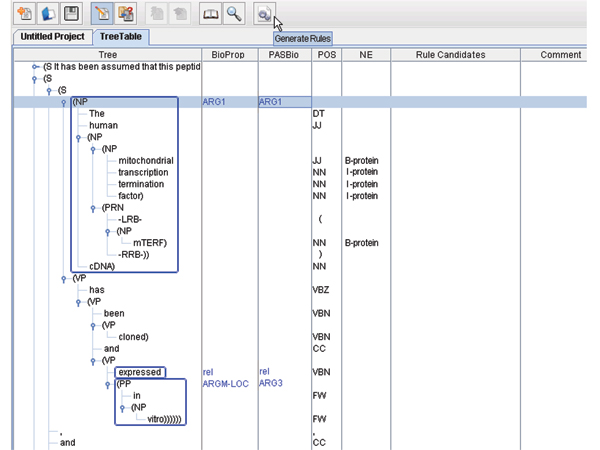
Screenshot of the rule-generation tool.

**Figure 3:**
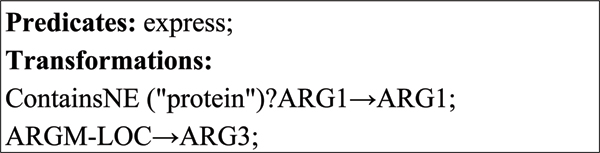
Conversion rule for the verb "express" for Figure 2.

Figure 2 shows a screenshot from the tool. The user feeds the tool with sentences containing PASBio-based semantic role information. The information is placed in the "PASBio" column after loading. The sentences are pre-processed to generate full parse tree structures, BioProp-based SRL, POS's, as well as NEs information represented in the first, second, fourth and fifth columns, respectively. After pre-processing, the tool allows users to view, modify or create conversion rules by clicking on the "Generate Rules" button as shown in Figure 2. A conversion rule generated after clicking the button is shown in Figure 3.

Each conversion rule consists of two elements: predicates and transformations. The predicate is the target verb. The first part of each transformation is the condition, which specifies the criteria that the arguments should follow. These criteria are defined as the composition of one or more logical predicates, which are concatenated by logical operators, such as AND, and OR. Two most common predicates are ContainsNE(*ne*) and ContainsKeywords(*kw*). The former is true if the argument contains at least one instance of the NE type *ne*. The latter is true if the argument contains at least one specified keyword *kw*. If there are no conditions for a transformation, this part can be omitted.

The second part is the mapping between a BioProp argument and a PASBio argument. The mapping consists of three elements: the source argument, an arrow "→", and the destination argument. For example, the transformation in Figure 3 defines a mapping from ArgM-LOC to Arg3. All the arguments that are not defined in the transformation source field are dropped.

As shown in Figure 3, the condition of the transformation "ARG1 → ARG1" is ContainsNE("protein"), which is interpreted as the mapping ARG1 → ARG1 holds if ARG1 contains at least one protein. For a case in which arguments match, such as that in Figure 3, the conversion rules can be automatically generated as follows:

1. For each argument pair, (*argument*_*B*_, *argument*_*P*_), if the argument phrase does not contain any recognized NEs, a simple rule will be generated in the argument's "Rule Candidates" field: *argument*_*B *_→ *argument*_*P*_

2. If the argument contains recognized NE types (*NE*_*type*_), they will become the conditions imposed on the argument, and the following rule type will be generated: ContainsNE (*NE*_*type*_)?*argument*_*B *_→ *argument*_*P*_

Users can modify the generated rules by editing the "Rule Candidates" field.

**Figure 4:**
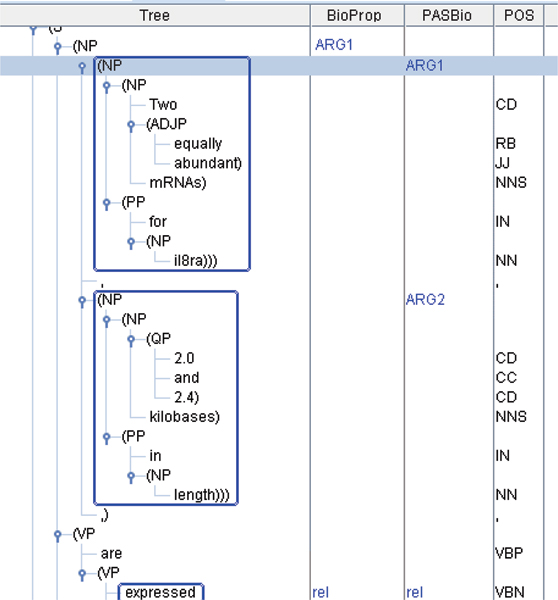
Multiple overlap for the verb "express".

In addition to defining simple conditions, such as ContainsNE, we also describe complex conditions using a format called the bracket form pattern, which can represent syntactic and semantic information as criteria. The pattern can be applied when two or more PASBio arguments are covered by only one BioProp argument (Figure 4), and vice versa. A bracket form [22] is a representation of a parse tree using brackets (Figure 4), to show the tree's structure.

A simplified bracket form for the parse tree shown in Figure 4, with some internal bracket divisions omitted for clarity: (NP (NP (Two equally abundant mRNAs for il8ra)) (,) (NP (2.0 and 2.4 kilobases in length))). 

Each constituent and its daughters are enclosed with brackets. If we replace constituent words in the phrase with a wildcard symbol "(.*)", the above bracket form becomes:

(NP (NP (.*)) (.*) (NP (.*)))

We can then use the bracket form as a pattern to match parse trees with the same structures.

To make these patterns more precise, we can add restrictions on the phrase constituents, such as limiting their semantic roles, head words and head words' UPENN POS [23]. To restrict a constituent's semantic role, one would insert a hyphen followed by the semantic role after the constituent type. For example, (NP) might become (NP-Arg1). The head word can be defined as the most important word in a constituent [24], and we identify it using Collins' [25] rule-based method. Head words of constituents are marked with an ampersand followed by the head word – e.g. (NP_@kilobase_). And the UPENN POS of the head word is placed directly after, separated by a forward slash – e.g. (NP_@kilobase/NNS_). If we combine our above examples, we can make the pattern, "(NP-Arg1@_mRNA/NNS _(NP_@mRNA/NNS _(.*)) (NP_@kilobase/NNS _(.*)))", where the outside NP must be Arg1, and the inside NPs' head word must be "mRNA" and "kilobase" with POS's "NNS."

In our notation, a rule will appear as follows:

BracketFormPattern(*x*) ? *C*_0 _→ *argument*_0_, *C*_1 _→ *argument*_1_,..., *C*_*i *_→ *argument*_*i*_,..., *C*_*k *_→ *argument*_*k*_;

"BracketFormPattern" is a logical predicate which means the source argument, *argument*_*s*_, must match the bracket form pattern *x *for the transformations "*C*_*i *_→ *argument*_*i*_" to occur, where *C*_*i *_is any constituent of a source argument annotated by PASBio.

In the example in Figure 4 for the verb "express", "ARG1" in the BioProp column does not directly match any one PASBio argument, but instead overlaps two arguments, Arg1 and Arg2. The rule-generation algorithm first generates two bracket forms for the unmatched noun phrase "Two equally abundant mRNAs for il8ra 2.0 and 2.4 kilobases in length", one for the "BioProp" column and the other for the "PASBio" column:

(NP-Arg1_@mRNA/NNS _(NP_@mRNA/NNS _(.*)) (.*) (NP_@kilobase/NNS _(.*))")

(NP_@mRNA/NNS _(NP-Arg1_@mRNA/NNS _(.*)) (.*) (NP-Arg2_@kilobase/NNS _(.*))")

Then, the first bracket form is merged with the second one as follows:

(NP-Arg1_@mRNA/NNS _(NP-*C*_0@mRNA/NNS _(.*)) (.*) (NP-*C*_1@kilobase/NNS _(.*))")

As you can see in the merged bracket form, all the PASBio constituents annotated with semantic roles are represented by the variable *C*_*i*_. For example Arg1 becomes *C*_0_.

Finally, the following three rules are automatically generated in the "Rule Candidates" field:

1. BracketFormPattern("(NP-Arg1 (NP-*C*_0 _(.*)) (.*) (NP-*C*_1 _(.*))") ? *C*_0 _→ Arg1, *C*_1 _→ Arg2

2. BracketFormPattern("(NP-Arg1_@mRNA/_(NP-*C*_0@mRNA/_(.*)) (.*) (NP-*C*_1@kilobase/_(.*))") ? *C*_0 _→ Arg1, *C*_1 _→ Arg2

3. BracketFormPattern("(NP-Arg1_@/NNS _(NP-*C*_0@/NNS _(.*)) (.*) (NP-*C*_1@/NNS _(.*))") ? *C*_0 _→ Arg1, *C*_1 _→ Arg2

The first rule is the loosest, only considering the parse tree structure and SRL tags. The second also considers the head word, and the third adds POS information as well. The user can check these rule candidates, and remove or modify the inappropriate ones.

Although these rules are semi-automatically generated, we have found from our observations that with slight human modification, they can be quite accurate. For the example in Figure 4, it is obvious that the first rule with no constraints on *C*_0 _and *C*_1 _is too loose. Likewise, the third rule, which limits *C*_0 _and *C*_1_'s POS to NNS, is too strict. However, the second rule is surprisingly accurate. If we look at the frameset definitions in BioProp and PASBio shown in Table 2, we can see that PASBio defines Arg2 as a property of Arg1 and limits Arg1 to a gene or gene product name. Therefore, if we wish to annotate *C*_0 _as Arg1 and *C*_1 _as Arg2, they must match these two conditions. Rule two stipulates that *C*_1_'s head word should be "kilobase" and *C*_0_'s should be "mRNA", which matches PASBio's frameset definition for "express" because "kilobase" is a unit of mRNA. Therefore, the annotator could choose the second rule with head word information.

**Table 2:** The frameset of the verb "express" in BioProp and PASBio

Predicate: express		
Argument	BioProp	PASBio

Arg0	causer of expression	no definition
Arg1	thing expressing	named entity being expressed
		//gene or gene products//
Arg2	end state	property of the existing named entity [Arg1]
Arg3	start state	location referring to organelle, cell or tissue

## Results

### Datasets

The training data of our SRL system, BIOSMILE, is an extended version of BioProp [13]. A total of 2,304 PAS's were annotated for 49 biomedical verbs. To evaluate BIOSMILE, the rule-based converter and the combined system, our in-lab biologists re-annotated the 313 annotated sentences available on PASBio's website according to the BioProp annotation format. The dataset from PASBio's website is hereafter referred to as PASBio_P _and the PASBio_P _dataset annotated using the BioProp format is referred to as PASBio_B_.

### Evaluation metrics

Performance was evaluated in terms of three metrics: precision (P), recall (R) and F-scores (F), which are defined as follows:

$$\begin{array}{c} \text{Precision}=\frac{\text{the number of correctly recognized arguments}}{\text{the number of recognized arguments}}\\ \text{Recall}=\frac{\text{the number of correctly recognized arguments}}{\text{the number of true arguments}}\\ \text{F}-\text{scores}=\frac{2\times \text{Precision}\times \text{Recall}}{\text{Precision}+\text{Recall}} \end{array}$$

For SRL and conversion evaluation, the official CoNLL-2004 [6] SRL evaluation script was used.

### BIOSMILE performance

We followed the same experimental procedure that we used in [14] to evaluate BIOSMILE performance on the extended BioProp dataset, details about which can be found in Additional file 1. The average results were an F-score of 72.67%, a precision of 81.72% and a recall of 65.42%.

To evaluate the actual performance on arbitrary sentences and verbs, we used PASBio_B _as an extra test data. BIOSMILE achieved an overall F-score of 67.31%, a precision of 76.28% and a recall of 60.22%. (More detailed performance data for each argument type can be found in Additional file 1.) The drop in BIOSMILE's performance on PASBio_B _may be caused by the following factor: Even though BioProp contains all PASBio verbs, it contains very few PAS's for some verbs, which likely decreases the accuracy of ML-based SRL on those verbs. For example, there is only one PAS for "splice" and two for "begin".

### Main system performance

We conducted two experiments – the first to test the BioProp-PASBio converter independently of BIOSMILE SRL performance, and the second to evaluate combined system performance. For both, 3-fold cross validation (CV) was applied, which involved partitioning the PASBio_p _dataset into three subsets. A single subset is retained as the test data, and the remaining two subsets are used as training data for generating conversion rules. The CV process is then repeated three times, with each of the test sets being used exactly once.

### Experiment 1: Evaluating the rule-based converter

In this experiment, we examined conversion performance using the PASBio_P _dataset, first feeding the PASBio_B _(gold-standard BioProp annotation) to the rule-based converter and then comparing the converted results with the PASBio_P _annotation to examine the precision, recall and F-scores. By using the PASBio_B_, we can effectively eliminate the influence of BIOSMILE SRL performance from this test. As shown in Table 3, we achieved an average F-score of 85.29%. The high F-score demonstrates the feasibility of our proposed semi-automatic conversion method.

**Table 3:** Rule-based converter performance (on PASBio_p_)

Argument Type	Precision	Recall	F-score
Arg0	86.36	92.36	89.26
Arg1	90.04	87.85	88.93
Arg2	88.03	70.55	78.33
Arg3	90.00	64.29	75.00
Arg4	66.67	54.54	60.00
ArgM-MNR	88.89	100.00	94.12
ArgM-MOD	100.00	100.00	100.00
ArgM-NEG	100.00	100.00	100.00
ArgR	75.00	33.33	46.15

Overall	88.55	82.27	85.29

### Experiment 2: Evaluating the combined system

In this experiment, we examined the combined performance of our system, as shown in Table 4. Compared with Experiment 1, the recall of the combined system drops 23%; however, the precision only drops 6%. This may be due to the fact that BIOSMILE has a high precision on PASBio_B _(76.28%) but a low recall (60.22%). In addition, comparing the results in Table 4 to the BIOSMILE performance on PASBio_B_, we can see that the combined system's performance is higher. This might seem counterintuitive; however, if we take into account that some argument types with low accuracy, such as ArgM-TMP and ArgM-DIR, are not converted to PASBio since PASBio does not define those arguments, then we can explain this discrepancy.

**Table 4:** Combined system performance

Argument Type	Precision	Recall	F-score
Arg0	79.49	64.58	71.26
Arg1	79.65	63.89	70.91
Arg2	87.80	49.32	63.16
Arg3	95.65	39.29	55.70
Arg4	100.00	45.45	62.50
ArgM-MNR	88.89	100.0	94.12
ArgM-MOD	100.00	100.00	100.00
ArgM-NEG	100.00	100.00	100.00
ArgR	100.00	22.22	36.36

Overall	82.85	59.23	69.08

## Discussion

After examining the PAS's which were not labeled correctly in the experiments, we have concluded that the following two factors affected conversion performance most strongly:

### Absence of key terms for argument disambiguation

In cases where one BioProp argument can be divided into two or more PASBio arguments, our rules may be insufficient to disambiguate if NEs or keywords are absent. Consider the following example annotated by our system with BioProp/PASBio annotations both given concatenated by a forward slash:

... [protein extracts from the transfected COS cells _**Arg0/Arg0**_] [inhibited _**V**_] [both the C alpha and C beta isoforms of the PKA catalytic subunit with equal efficacy _**Arg1/Arg2**_].

The last argument is incorrectly converted from BioProp Arg1 to PASBio Arg2 by our system. To find out why, we must look at BioProp and PASBio's frameset definitions for "inhibit" shown in Table 5.

**Table 5:** The frameset of the verb "inhibit" in PASBio and BioProp

Argument	BioProp
Arg0	Inhibitor
Arg1	entity inhibited

Argument	PASBio

Arg0	agent
Arg1	the entity being inhibited by agent to get binding
Arg2	the action or property being inhibited

We can see that PASBio defines both Arg1 and Arg2 as the objects being inhibited, but Arg1 is further constrained to being the entity bound by the agent. BioProp, which has no Arg2 definition, does not make this distinction. The automatically generated conversion rule for Arg1, therefore, will have the constraint ContainsKeywords("binding"). However, as the above example lacks any references to binding that would describe which entity "gets binding", the system converts to Arg2 instead of Arg1. In this case, simple NE-/keyword-based rules cannot distinguish Arg1 from Arg2.

According to our analysis, 3.83% of the PAS's in the PASBio_P _suffered from this problem, especially PAS's for verbs such as decrease, delete, inhibit, lost, mutate, transcribe and truncate.

### Coordination ambiguity

Coordination ambiguity in the full parse information is another factor that affects conversion performance.

**Figure 5:**
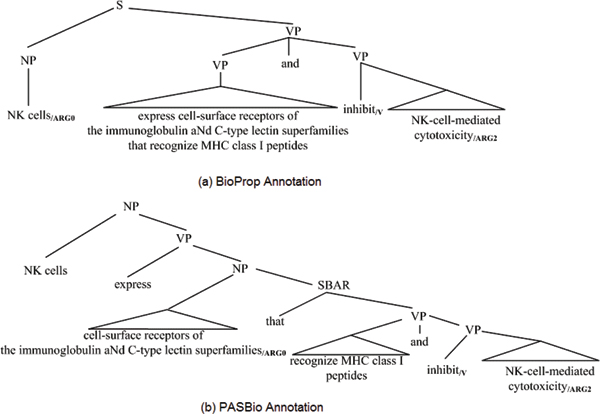
Coordination ambiguity.

Figure 5 shows two possible full parse structures for the following sentence:

NK cells express cell-surface receptors of the immunoglobulin and C-type lectin superfamilies that recognize MHC class I peptides and inhibit NK-cell-mediated cytotoxicity.

The phrase "inhibit NK-cell-medidated cytotoxicity" can be coordinated with three different phrases, each with a different meaning. This syntactic ambiguity is referred to as "coordination ambiguity" [25] and is a major problem in parsing. As you can see in Figure 5(a), our full parser coordinates the verb phrase "express cell-surface receptors of the ... class I peptides" with the verb phrase "inhibit NK-cell-mediated cytotoxicity." Therefore, BIOSMILE tags the noun phrase "NK cells" as "Arg0" for the verb "inhibit." However, in the gold standard annotation, the PASBio developers annotate the "cell-surface receptors of ... superfamilies" as "Arg0" for the verb "inhibit". The parse tree for the PASBio's annotation is illustrated in Figure 5(b). It coordinates the verb phrase "recognize MHC class I peptides" with the verb phrase "inhibit NK-cell-mediated cytotoxicity." Although, both these parse trees were generated by our parser initially, in the end, it chose the incorrect one, Figure 5(a), because, based on the training data, that one appeared to have the highest probability. In such cases it is impossible to distinguish the correct choice using syntactic parsing. Our results show that 1.92% PAS's in the PASBio_P _dataset suffered this problem.

### Correlation between BIOSMILE and combined system performance

**Figure 6:**
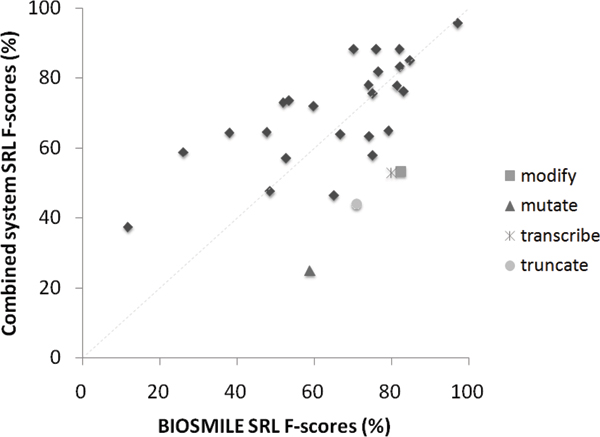
Correlation between BIOSMILE and combined system performance.

Figure 6 shows a scatter diagram which plots BIOSMILE's SRL F-score against the combined system's. Each data point represents one PASBio verb. The correlation between these two F-scores is 0.52, which is in the range of moderately positive correlation (0.4–0.7). We examined the outlying verbs with the greatest drops in F-score after conversion. These included "mutate", "truncate", "transcribe", and "modify". We found that the first three suffered from an absence of key terms. The last verb, modify, had less than five annotated sentences in the PASBio_P _corpus, making it difficult for our algorithm to generate effective transformation patterns.

## Conclusion

In this paper we have demonstrated the feasibility of converting between BioProp and PASBio annotation, which will hopefully facilitate and inspire further PASBio applications. Our approach has involved the use of our previous SRL system, BIOSMILE, as well as the development two new tools, a semi-automatic rule generator and a BioProp-PASBio converter. Our rule-generation tool can save considerable human effort by automatically generating conversion rules which only need fine tuning to be usable. Our BioProp-PASBio converter can achieve very high accuracy (85.29%) using the gold-standard BioProp dataset. Our combined system (BIOSMILE + rule-based converter) achieves an F-score of 69.08% for PASBio's 29 verbs. This performance is close to state-of-the-art ML-based SRL systems in other specific domains [26].

## Competing interests

The authors declare that they have no competing interests.

## Authors' contributions

RTH Tsai and HJ Dai designed the semi-automatic rule generation and rule-based conversion algorithms and wrote most of this paper. HJ Dai implemented the conversion algorithm and wrote the rule generator and rule-based converter program. CH Huang, the biologist in our laboratory, verified the generated rules and conducted all experiments. RTH Tsai and WL Hsu guided the whole project.

## Supplementary Material

Additional file 1 Supplementary materials. Click here for file
