# Supplementary material for: Semi-automatic conversion of BioProp semantic annotation to PASBio annotation
Source: BMC Bioinformatics. 2008 Dec 12;9(Suppl 12):S18. doi: 10.1186/1471-2105-9-S12-S18 (PMC2638158; doi:10.1186/1471-2105-9-S12-S18)
Supplement: Additional file 1 — Supplementary materials. [file 1471-2105-9-S12-S18-S1.pdf]

Supplementary materials

The definitions of the argument types in PropBank

**Type**	**Definition**
Arg(n)	Main arguments whose definitions depend on the corresponding predicate
ArgM-DIR	Directional modifiers show motion along some path
ArgM-LOC	Locative modifiers indicate where some action takes place
ArgM-MNR	Manner adverbs specify how an action is performed
ArgM-EXT	Extent markers indicate the amount of change occurring from an action
ArgM-REC	Reciprocals markers include reflexives and reciprocals
ArgM-PRD	Markers of secondary predication, these are used to show that an adjunct of a predicate is in itself capable of carrying some predicate structure
ArgM-PNC	Purpose clauses are used to show the motivation for some action
ArgM-CAU	Cause clauses, similar to "Purpose clauses", these indicate the reason for an action
ArgM-DIS	Discourse markers, these are markers which connect a sentence to a preceding sentence
ArgM-ADV	Adverbials, these are used for syntactic elements which clearly modify the event structure of the verb in question, but which do not fall under any of the argument types above
ArgM-NEG	Negation, this tag is used for elements such as "not", "n't", "never", "no longer" and other markers of negative sentences
ArgM-MOD	Modals are: will, may, can, must, shall, might, should, could, would. Phrasal modals such as "going (to)", "have (to)" and "used (to)" are also included
ArgM-TMP	Temporal markers show when an action took place

Distribution of PASBio restrictive framesets

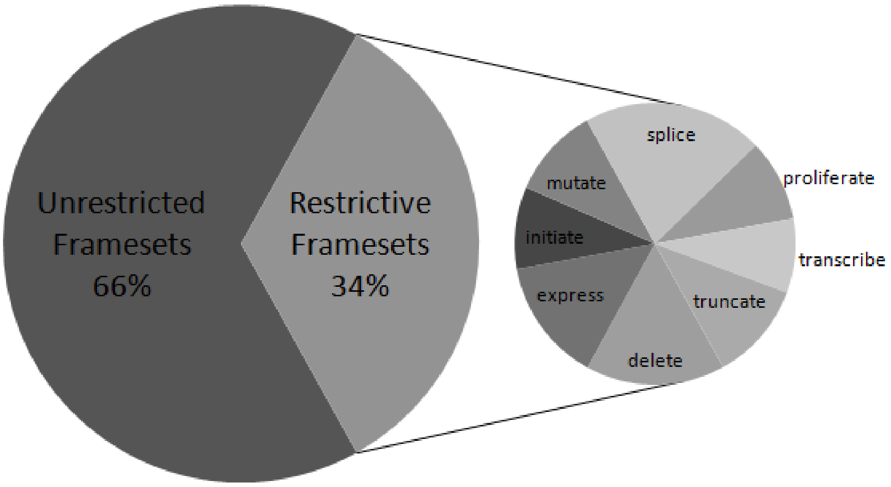

Figure 1 - Distribution of restrictive framesets

Figure 1 shows the distribution of restrictive framesets among biomedical verbs in the 313 PAS annotated example sentences available on PASBio's website.

The Backus-Naur form for the conversion rules

```
<statement>
        ::=   <predicate_statement>   <transformation_statement>
        |     ";"

<predicate_statement> ::= "Predicates: "<string_list> ";"

<string_list> ::= <string_expression>

<string_expression> ::= string

<transformation_statement> ::= "Transformations: " <conversion_expression>

<conversion_expression> ::= {{<expression> "?"} <expression> ";"}

<expression>
        ::= <argument_identifier>
        |   <predicate_expression>
        |   <logical_expression>
        |   <transformation_expression>

<transformation_expression>
        ::= <expression> "→" <expression> "

<logical_expression>
        ::= "NOT" <expression>
        |   <expression> <logical_operator> <expression>
        |   "true"
        |   "false"

<logical_operator>
        ::= "AND" | "OR" | ","

<argument_identifier> ::= "ARG"<argument_type>

<argument_type> ::= "0" | "1" | "2" | "3" | "4" | "R" | "M-LOC" | "M-MNR" | "M-ADV" |
"M-NEG" | "M-TMP" | "M-EXT" | "M-PNC" | "M-CAU" | "M-DIR" | "M-DIS" | "M-MOD"
| "M-REC" | "M-PRD"

<predicate_expression> ::= <predicate_identifier>"(" [<parameter_list>] ")"

<predicate_identifier> ::= "A..z, $, _"{ "A..z, $, _, 0..9" }

<parameter_list> ::= <parameter> {"," <parameter>}

<parameter>
        ::= <string_list>
        |   <argument_identifier>
        |   <predicate_expression>
```

Full parser performance

Lease and Charniak [1] adapted a newswire full parser to the biomedical field in 2005 which used the Penn Treebank [2] as its training set and the GENIA Treebank (GTB) 200, which contains 200 abstracts (1,732 sentences) released by Tsujii laboratory on Sep. 22, 2004, as its test set. We use the same test set, GTB 200, but replace the training data with the GTB 300 which contains 300 non-overlapped abstracts (2,768 sentences) released on July 11th, 2005. Our parser achieved an F-score of 85% versus Lease's 82.9% by using the standard PARSEVAL evaluation script [3]. Our parser also outperforms the other eight biological parsers reported by Clegg and Shepherd [4].

BIOSMILE performance

We trained BIOSMILE system on 30 randomly selected training sets from BioProp, each having 1,700 training PAS’s with the restriction that the parse tree information must have been generated by our full parser (the performance is reported in the "Full parser performance" section). After the training process, we tested our system on 30 600-PAS test sets, each of which is independent from the corresponding training set. We then summed the scores and calculated the averages to get the evaluation results; the precision, recall and F-score are 81.72, 65.42 and 72.67%, respectively.

To evaluate the actual performance on arbitrary sentences and verbs, we used the PASBio-EX as extra test data. The following table shows detailed results for each argument.

BIOSMILE performance on PASBio-EX dataset

Argument Type	Precision	Recall	F-score
Arg0	70.35	65.76	67.98
Arg1	82.83	64.55	72.56
Arg2	84.38	47.37	60.67
ArgM-ADV	42.86	38.71	40.68
ArgM-DIR	100.00	19.05	32.00
ArgM-DIS	78.95	78.95	78.95
ArgM-LOC	68.97	37.04	48.19
ArgM-MNR	87.80	56.25	68.57
ArgM-MOD	86.96	95.24	90.91
ArgM-NEG	100.00	100.00	100.00

ArgM-TMP	46.67	41.18	43.75
R-Arg0	75.00	87.50	80.77
R-Arg1	54.55	46.15	50.00
Overall	76.28	60.22	67.31

Extended BioProp corpus

30 verbs selected by Chou et al.		Extra 19 verbs selected from PASBio	
activate	mediate	abolish	result
affect	modulate	begin	splice
alter	mutate	catalyse	transcribe
associate	phosphorylate	confer	truncate
bind	prevent	delete	
block	promote	develop	
decrease	reduce	disrupt	
differentiate	regulate	eliminate	
encode	repress	generate	
enhance	signal	initiate	
express	stimulate	lead	
increase	suppress	lose	
induce	transactivate	modify	
inhibit	transform	proliferate	
interact	trigger	recognize	

References

1. Lease M, Charniak E: **Parsing biomedical literature**. *Second International Joint Conference on Natural Language Processing* 2005:58–69.
2. Marcus MP, Santorini B, Marcinkiewicz MA: **Building a Large Annotated Corpus of English: The Penn Treebank**. *Computational Linguistics* 1994, **19**:313-330.
3. Black E, Abney S, Flickenger D, Gdaniec C, Grishman R, Harrison P, Hindle D, Ingria R, Jelinek F, Klavans J: **A procedure for quantitatively comparing the syntactic coverage of English grammars**. *Proceedings of the February 1991 DARPA Speech and Natural Language Workshop* 1991, **10**.
4. Clegg A, Shepherd A: **Benchmarking natural-language parsers for biological applications using dependency graphs**. *BMC Bioinformatics* 2007, **8**(1):24.
